# Overworked and Underpaid Hospital Nurses

**Published:** 1918-12-21

**Authors:** J. P. Lucas

**Affiliations:** Yelverton, Devon


### THE EDITOR'S LETTER-BOX.

**[Correspondence on all subjects is invited, but we cannot in any way be responsible for the opinions expressed by our correspondents, who must give their name and address as a guarantee of good faith, but not necessarily for publication. Correspondents are reminded that brevity of style and conciseness of statement greatly facilitate early insertion.]**

### Overworked and Underpaid Hospital Nurses.

*To the Editor of* THE HOSPITAL.

SIR,—The College of Nursing deserves well for its decisive action in urging its members to profit by the General Election to endeavour to obtain proper Parliamentary support for the College Bill; the more so, as this will further extend influence in other directions, and be of material assistance to obtain for hospital nurses the just reforms so pressingly needed in regard to their hours of duty, as also their pay.

The election so far as actual polling is concerned is over, and it is understood that the women voters made the fullest use of their political rights; but it is still necessary to urge those nurses who recorded their vote, and the parents and relatives of those who had not this right, to keep in touch with their newly-elected Members of Parliament until such measures are introduced as will remove from our midst the scandal of hospital nurses being overworked and underpaid.

To rely upon hospital boards to bring these reforms is futile. After protracted correspondence with the chairman of one of the leaders—holding also multiple office—I am convinced that even the need for reform is not generally admitted. It may be, and probably is, that financial considerations compel an ambiguous attitude, but if so, the greater the urgency for a State-subsidy so that the subject may be approached on its actual merits alone.

The time has come, too, for nurses, who hitherto have conspicuously failed to do so, to assert themselves, and their right to shorter hours, and to compel the attention of Parliament to the unfair and unequal conditions under which they labour, for the great majority of them have adopted professional nursing as the means of a living, and at present their calling is by almost common assent the most overworked and underpaid in existence.

Speaking at Plymouth last week, Dr. Mabel Ramsay strongly advocated the cause of army and civilian nurses, describing both as scandalously underpaid, as to the accuracy of which statement there should be no divergence of opinion; and doubtless the same views were expressed elsewhere, but unfortunately are not reported; it is thus to be regretted that the general public remain to a large extent in ignorance of the facts, and to overcome this the sympathetical aid of the Press never withheld in a good cause should be invoked.—Yours obediently,

J. P. LUCAS.

Yelverton, Devon, December 16.

